# Identification of the Transcriptional Networks and the Involvement in Angiotensin II-Induced Injury after CRISPR/Cas9-Mediated Knockdown of Cyr61 in HEK293T Cells

**DOI:** 10.1155/2019/8697257

**Published:** 2019-04-15

**Authors:** Junjie Wang, Dongdong Fu, Soulixay Senouthai, Yan Jiang, Rentong Hu, Yanwu You

**Affiliations:** ^1^Department of Nephrology, Affiliated Hospital of Youjiang Medical University for Nationalities, Baise, Guangxi Zhuang Autonomous Region, China; ^2^Department of Clinical Laboratories, Affiliated Hospital of Youjiang Medical University for Nationalities, Baise, Guangxi Zhuang Autonomous Region, China; ^3^Science Lab Center, Youjiang Medical University for Nationalities, Baise, Guangxi Zhuang Autonomous Region, China

## Abstract

**Background:**

The transcriptional networks of Cyr61 and its function in cell injury are poorly understood. The present study depicted the lncRNA and mRNA profiles and the involvement in angiotensin II-induced injury after Cyr61 knockdown mediated by CRISPR/Cas9 in HEK293T cells.

**Methods:**

HEK293T cells were cultured, and Cyr61 knockdown was achieved by transfection of the CRISPR/Cas9 KO plasmid. lncRNA and mRNA microarrays were used to identify differentially expressed genes (DEGs). Gene ontology (GO) and the Kyoto Encyclopedia of Genes and Genomes (KEGG) pathway analyses were performed to determine biofunctions and signaling pathways. RT-PCR was used to validate the microarray results. Cells were divided into four groups: control, Cyr61 knockdown, angiotensin II (Ang II) without Cyr61 knockdown, and Ang II with Cyr61 knockdown. CCK8, western blotting, and flow cytometry analysis were carried out to dissect cellular function.

**Results:**

A total of 23184 lncRNAs and 28264 mRNAs were normalized. 26 lncRNAs and 212 mRNAs were upregulated, and 74 lncRNAs and 233 mRNAs were downregulated after Cyr61 knockdown. Analysis of cellular components, molecular functions, biological processes, and regulatory pathways associated with the differentially expressed mRNAs revealed *down*stream mechanisms of the Cyr61 gene. The differentially expressed genes were affected for small cell lung cancer, axon guidance, Fc gamma R-mediated phagocytosis, MAPK signaling pathway, focal adhesion, insulin resistance, and metabolic pathways. In addition, Cyr61 expression was increased in accordance with induction of cell cycle arrest and apoptosis and inhibition of cell proliferation induced by Ang II. Knockdown of Cyr61 in HEK293T cells promoted cell cycle procession, decreased apoptosis, and promoted cell proliferation.

**Conclusions:**

The Cyr61 gene is involved in Ang II-induced injury in HEK293T cells. Functional mechanisms of the differentially expressed lncRNAs and mRNAs as well as identification of metabolic pathways will provide new therapeutic targets for Cyr61-realated diseases.

## 1. Introduction

Cysteine-rich protein 61 (Cyr61), also known as CCN1, was identified as secreted protein members of the CCN family. Cyr61 which is produced and secreted by endothelial cells, fibroblasts, and smooth muscle cells is a component of the extracellular matrix [1]. Cyr61 has the ability to regulate multiple signaling pathways and play an important role in different biological processes. *In vitro*, CCN proteins regulate the functions of cells, such as adhesion, migration, proliferation, differentiation, and survival, as well as induce cell apoptosis and extracellular matrix remodeling of different cell types. *In vivo*, it also plays a vital role in vascular and bone development and angiogenesis [2]. Furthermore, Cyr61 has highlighted the pivotal role this molecule can play in regulating the immune-surveillance process and it has emerged as an important partner when targeting components of the infectious or chronic inflammatory disease processes such as atherosclerosis or rheumatoid arthritis [3–5]. Additionally, several lines of evidence have shown that overexpression of Cyr61 is involved in the cancer process such as osteosarcoma [6], ovarian tumor [7], and breast adenocarcinoma [8]. Interestingly, Cyr61 was also found to be downregulated in prostate cancer and non-small cell lung carcinoma [9, 10]. This can be ascribed to its direct binding to different integrins in different cell types and a variety of environments [11]. Hence, Cyr61 may play a pivotal role and serve as a potential prognostic indicator or therapeutic target in a variety of diseases.

The mammalian genome is pervasively transcribed, producing a large number of noncoding RNAs (ncRNAs), including long noncoding RNAs (lncRNAs), which are defined as transcripts longer than 200 nucleotides [12, 13]. Studies have found that only one-fifth of transcription across the human genome is associated with protein-coding genes, indicating that there are at least four times more long noncoding than coding RNA sequences [14]. lncRNAs play an important role in the regulation of thousands of protein-encoding genes via various mechanisms, although these are not translated into proteins [15]. Transcriptome profiling has been carried out in various genes and conditions in order to characterize their functions and underlying mechanisms [16–19]. Unfortunately, studies of Cyr61 functional recovery have not yet been able to highlight its transcriptional networks. No study so far has reported using the gene chip array to find out about the differentially expressed lncRNAs and mRNAs associated to Cyr61.

As one of the earliest and most extensively studied hormonal systems, the renin-angiotensin system (RAS) which is involved in many physiological and pathological processes is an atypical hormonal system in several ways [20, 21], and the new components and functions of the RAS have still not been unraveled [22]. The RAS has powerful effects including the control of blood pressure and sodium homeostasis as well as fluid through integrated actions in the kidney, the cardiovascular system, and the central nervous system. Along with its impact on blood pressure, the RAS also affects others pathological processes including inflammation and immune responses [23]. Research on the RAS has contributed significantly to advances in the understanding and treatment of cardiovascular diseases [24]. As one of the major bioactive products of the RAS, angiotensin II (Ang II) has a well-known role in cardiovascular regulation and is a key regulator of renal inflammation and fibrosis as well as blood pressure and renal hemodynamics [25–27]. Several pathological effects are caused by the Ang II-induced TGF-*β* pathway which is a potent inflammatory as well as fibrotic and apoptotic cytokine [28, 29]. In addition, Ang II participates in cell proliferation, matrix degradation, inflammation, and apoptosis by activating a multiplicity of signaling pathways [30, 31].

In this study, the lncRNA and mRNA profiles were determined after Cyr61 knockdown mediated by CRISPR/Cas9 in HEK293T cells. The signal transduction and metabolic pathways associated with these differentially expressed lncRNA and mRNA were analyzed. The effect of Cry61 on Ang II-induced cell injury in HEK293T cells was also analyzed. The results provide the functional mechanisms of Cry61 as well as potential new targets for Cyr61-associated diseases.

## 2. Materials and Methods

### 2.1. Cell Lines and Cell Culture

The HEK293T cell line (which was purchased from the Molecular Microbiology and Immunology, Keck School of Medicine, University of Southern California) was cultured in Dulbecco's modified Eagle's medium (DMEM, Gibco, Su Zhou, USA) in the presence of 10% fetal bovine serum (FBS, Lanzhou Minhai Bio-Engineering, Gansu, China). All of the cells were maintained at 37°C in a humidified atmosphere with 5% CO_2_.

### 2.2. Knockdown of Cyr61 Gene Expression

The guide RNA sequences used are shown in Table 1. Cyr61 CRISPR/Cas9 KO plasmid, homology-directed repair (HDR) transfection plasmid, and UltraCruz Transfection Reagent were purchased from Santa Cruz Biotechnology (USA), and gene knockdowns were performed according to the manufacturer's protocol. The HEK293T cells were seeded in 6-well plates at 1.5 × 10^5^ − 2.5 × 10^5^ cells/3 mL per well, and the serum-free medium was changed after 70% confluence was achieved by routine culture. 1 *μ*g of each of Cyr61 CRISPR/Cas9 KO plasmid and HDR transfection plasmid and 10 *μ*L of UltraCruz reagents were added to 300 *μ*L of serum-free and antibiotic-free medium and in 6-well plates for incubation at room temperature for 10 min, followed by addition of antibiotic-free 10% FBS-containing media to a final volume of 2 mL. Wild-type HEK293T cells were treated with different concentrations of puromycin, and the results showed that the lowest inhibitory concentration to the HEK293T cells after 7 d of treatment was 8 *μ*g/mL and this concentration was subsequently used for screening. At 48 h posttransfection, the culture medium was replaced with 8 *μ*g/mL puromycin-containing DMEM medium for screening the cells.

### 2.3. Classification of Cells

The HEK293T cells were divided into four groups: (1) control group, nontransfected cells, (2) Cyr61-downregulated group, the third generation incubated after transfection, (3) Ang II group, nontransfected cells treated with Ang II (10^−7^ mol/L), and (4) Cyr61 downregulated + Ang II group, transfected cells treated with Ang II (10^−7^ mol/L). *Cyr61* expression in the control group and Cyr61-downregulated group were identified by western blotting, and these were subjected to microarray analysis. Cell proliferation, apoptosis, and cell cycle assays were performed for all the groups.

### 2.4. RNA Extraction and Quality Control

Total RNA was extracted from each sample by soaking the samples in TRIzol Reagent (Invitrogen, Grand Island, NY, USA) in accordance with the manufacturer's instructions. RNA quantity and quality were measured using a NanoDrop ND-1000, and RNA integrity was assessed by standard denaturing agarose gel electrophoresis. RNA quality test results are shown as Supplemental Material (Table 2). For spectrophotometer analysis, only samples with OD_260_/OD_280_ratio ≥ 1.8 and OD_260_/OD_230_ratio ≥ 1.5 were acceptable for further analysis.

### 2.5. Identification of Differentially Expressed lncRNAs and mRNAs

The mRNA-lncRNA Affymetrix microarrays (raw data) were used in this study. Limma (linear models for microarray data) package in R was used to identify differentially expressed lncRNAs and mRNAs between the 2 groups through use of the *t*-test. Fold change > 1 and *P* < 0.05 were regarded as the criteria for differential expression.

### 2.6. Gene Ontology (GO) and Pathway Enrichment Analysis

GO analysis is frequently used in functional enrichment studies of large-scale genes. Kyoto Encyclopedia of Genes and Genomes (KEGG) enrichment analysis was performed to analyze the biological pathways, involving the differentially expressed mRNAs. In the current study, DAVID (the database for Annotation Visualization and Integrated Discovery) software was used to investigate the functional enrichment condition for the up- and downregulated differentially expressed mRNAs. *P* < 0.05 was selected as the threshold.

### 2.7. Quantitative Real-Time PCR Validation

To validate the microarray data, we selected the top 3 most significant upregulated (FRMD1, SERPINF1, and FEZ1) and the top 3 most significant downregulated (PRR21, REG3G, and ACAT2) mRNAs as well as the top 3 most significant upregulated (RP11-659F24.1, RP11-966I7.4, and LAMB2P1) and the top 3 most significant downregulated (ANKRD30BL, CH17-360D5.2, and SOX2-OT) lncRNAs from mRNAs and lncRNAs that were expressed aberrantly. Briefly, 2 *μ*g of total RNA from each sample was used for the synthesis of first-strand cDNA using a FastKing RT Kit (Tiangen, Beijing, China) according to the manufacturer's protocol. Following first-strand cDNA synthesis, a PCR reaction was carried out in a 20 *μ*L reaction volume containing ×1 SuperReal PreMix Plus, 0.6 *μ*L of each specific forward and reverse primer, and 2 *μ*L of cDNA template. PCR assays were set at an initial denaturation step at 95°C for 3 min, followed by 40 cycles of 95°C for 10 s and 60°C for 30 s in a LightCycler® 96 system (Roche, Switzerland). Relative changes in gene expression were calculated using the 2^-∆∆Ct^ method with glyceraldehyde 3-phosphate dehydrogenase (GAPDH) as a reference gene as described previously [32, 33]. Each qPCR was carried out in triplicate, with each triplicate data point repeated 3 times. For the triplicates, only samples that differed by <0.3 Ct values were used in the final calculations. Less than about 5% of samples fell outside this range. In addition, standard curves were generated for each gene using a 50-fold dilution range. The data shown represented the means of three experiments. The primers for each gene are listed in Table 3.

### 2.8. Western Blotting Analysis

The HEK293T cells were harvested and washed twice with PBS, lysed in ice-cold radio-immunoprecipitation assay buffer (RIPA, Beyotime Biotechnology, Shanghai, China) with a freshly added 0.01% protease inhibitor cocktail (CWBiotech, Beijing, China), and incubated on ice for 30 minutes. Protein concentration was measured by bicinchoninic acid (BCA) protein assay kit (Beyotime Biotechnology, Shanghai, China). A total of 50 *μ*g of protein was subjected to electrophoresis using SDS-PAGE and transferred by electrophoresis onto a nitrocellulose membrane (GE Healthcare, Freiburg, Germany). Blots were visualized using enhanced chemiluminescence (ECL, Millipore, Billerica, MA, USA) after antibody binding. The antibody against Cyr61 was purchased from Santa Cruz Biotechnology (USA); antibodies against GAPDH were purchased from Cell Signaling Technology Biotech (Danvers, MA, USA). The bands were quantified by their densitometry with ImageJ software (NIH, USA).

### 2.9. Cell Proliferation Assay

Cell proliferation was performed using Cell Counting Kit-8 (CCK-8, Solarbio Life Sciences, Beijing, China) assay. The HEK293T cells were seeded in 96-well plates at a density of 5 × 10^3^ cells per well and cultured at 37°C in 5% CO_2_ for 12 hours and treated with Ang II (10^−7^ mol/L) [34]. After incubation for 0, 12, 24, and 48 h, 10 *μ*L CCK-8 solution was added to each well and incubated for another 1 to 4 hours. The absorbance was measured in the multimode microplate reader TriStar LB 941 (Berthold Technologies, Bad Wildbad, Germany) at 450 nm. Each experiment was performed in triplicate.

### 2.10. Cell Apoptosis Assay

Apoptosis was determined by using a flow cytometer (FACSCanto II, BD Biosciences, San Jose, CA, USA), and an Annexin V fluorescein isothiocyanate (FITC)/propidium iodide (PI) double-stain assay was performed in accordance with the manufacturer's protocol (FITC Annexin V/PI, BD Biosciences, San Diego, USA). After incubation for 48 hours, each supernatant was collected in a centrifuge tube and each group of cells was washed three times with phosphate-buffered saline (PBS), trypsinized, centrifuged (400 × *g* at room temperature) for 5 min, adjusted to 5 × 10^4^ cells/mL, and suspended in binding buffer containing Annexin V-FITC and PI. After incubation for a further 15 min at room temperature in the dark, the fluorescent intensity was measured using a flow cytometer. Each experiment was performed in triplicate.

### 2.11. Cell Cycle Assay

For cell cycle analysis, each group of cells was harvested after 48 h. Cell cycle analysis was conducted with the cell cycle detection kit (KeyGen, China) according to the manufacturer's instructions. Briefly, cells were harvested by regular trypsin digestion and rinsed with PBS. After fixation in 70% ethanol overnight at -20°C, cells were rinsed again in PBS and stained in a staining solution containing PI and RNase A. Flow cytometry (Canto II, BD Biosciences, USA) was employed to analyze the cellular DNA content. Each treatment was performed in triplicate.

### 2.12. Statistical Analysis

The results are presented as the mean value ± S.D. using SPSS23. One-way analysis of variance (ANOVA) was used among diverse groups, and independent samples were analyzed by Student's *t*-test when appropriate. A value of *P* < 0.05 was regarded as statistically significant.

To determine if DEGs overlapped with or were significantly enriched with a specific gene set, 28264 expressed genes in our samples were used as background for gene ontology (GO) and Kyoto Encyclopedia of Genes and Genomes (KEGG) pathway analyses assessed by DAVID.

## 3. Results

### 3.1. The Expression Levels of Cyr61 Protein in HEK293T Cells

**Figure 1:**
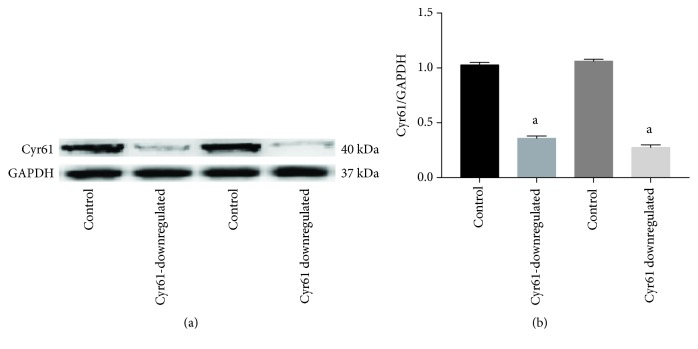
The expression of Cyr61 protein by transfection of CRISPR/Cas9 KO plasmid. The expression of Cyr61 protein was confirmed by western blotting. The Cyr61 protein expression levels were significantly decreased in HEK293T cells where the Cyr61 gene was knocked down when compared with those of the controls (^a^*P* < 0.05). The data represent mans ± SD from 3 independent experiments.

To examine the biological function of Cyr61 in HEK293T cells, the expression of Cyr61 was knocked down in HEK293T cells by the CRISPR/Cas9 KO plasmid, and three separate CRISPR gRNA sequences targeted to the Cyr61 gene were designed. The HEK293T cells were transfected with CRISPR/Cas9 vectors containing each of the three target sequences. After screening by puromycin, one homozygous was found by western blotting analysis. As shown in Figure 1, the Cyr61 protein expression levels were significantly decreased in HEK293T cells from 1.03 ± 0.024 to 0.36 ± 0.021 and from 1.06 ± 0.018 to 0.28 ± 0.023 (Cyr61/GAPDH, respectively) after knockdown from control levels.

### 3.2. Differential Gene Expression Analysis between Experimental and Control Groups

**Figure 2:**
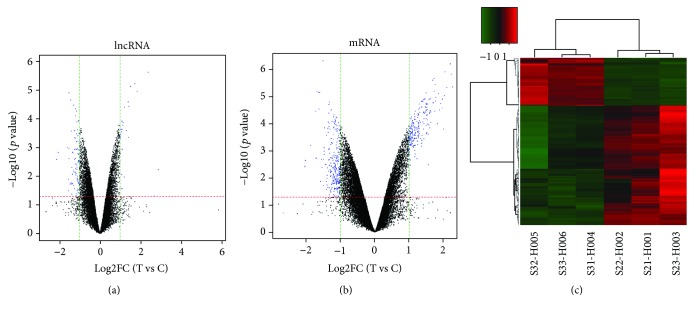
Volcano plots and heat map. Volcano plots of all the detected lncRNAs (a) and mRNAs (b) in the control group and downregulated Cyr61 group. The red- and green-dotted lines represent *P* values and multiple screening thresholds, respectively. The blue plots represent significantly changed genes with ≥1.0-fold change and *P* values of 0.05. The heat map displayed differentially expressed mRNAs. (c). Each column represents a sample, and each row represents a dysregulated RNA transcript. The red and green stripes imply that the RNA was upregulated and downregulated, respectively, in the downregulated Cyr61 sample.

100 differentially expressed lncRNAs with a fold change cutoff of 1.0 (26 upregulated and 74 downregulated, *P* < 0.05) were identified from the lncRNA microarrays of the Cyr61 experimental and control groups. Volcano plots were constructed to identify differences in the lncRNAs. The top 10 most significant up- and downregulated lncRNAs are shown in Table 4. RP11-659F24.1 and ANKRD30BL were the most up- and downregulated lncRNA transcript with a fold change of 2.87 and 2.13, respectively. The volcano plots display the expression profiles for all detected lncRNAs (Figure 2(a)).

446 differentially expressed mRNAs were also identified by using mRNA microarrays with a fold change cutoff of 1.0 (212 upregulated and 223 downregulated, *P* < 0.05) in Cyr61 knocked down HEK293T cells when compared with controls. The top 10 most significant up- and downregulated mRNAs are shown in Table 5. FRMD1and PRR21 were the most up- and downregulated mRNA transcripts with the fold change of 2.7 and 2.02, respectively. The volcano plots display the expression profiles for all detected mRNAs (Figure 2(b)). The heat map is shown in Figure 2(c).

### 3.3. Gene Ontology Analysis

**Figure 3:**
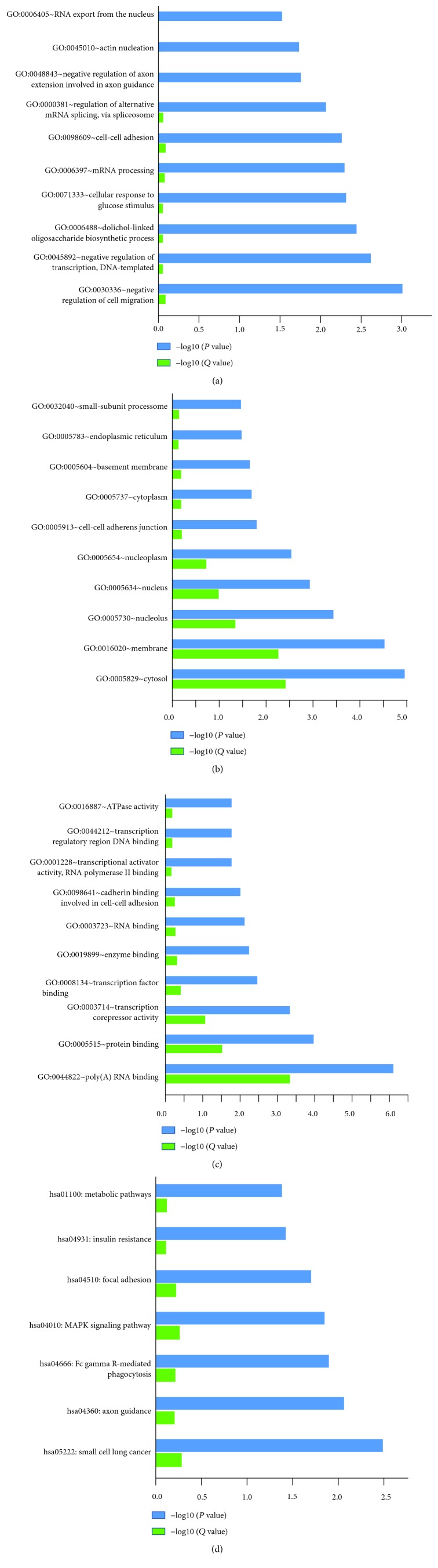
Pathways and summary of GO terms. Enriched GO terms by DAVID (top) and pathway by KEGG (a). The top ten GO enrichment result of biological process (b). The top ten GO enrichment result of cellular component (c). The top ten GO enrichment result of molecular function (d). Enrichment of biological pathway. *P* values were corrected by the fit linear model.

The 446 differentially expressed mRNAs underwent Gene Ontology (GO) analyses by using the DAVID software. GO analysis of the differentially expressed genes was performed to determine the gene product attributes in biological processes, cellular components, and molecular functions. *P* < 0.05 denoted the significance of GO term enrichment in the differentially expressed genes, and the lower the *P* value, the more significant the GO term. The top 10 biological processes with the highest *P* values are listed in Table 6 and Figure 3(a), and the most significant process is related to “regulation of cell migration.” The top 10 cellular components with the highest *P* values are shown in Table 7 and Figure 3(b), and the most significant one is the “cytosol.” The top 10 molecular functions terms with the highest *P* values are displayed in Table 8 and Figure 3(c), and the most significant one is “poly (A) RNA binding.”

### 3.4. KEGG Pathway Analysis

Pathway analysis is a functional analysis that maps genes to the KEGG pathways. The target genes for differentially expressed mRNAs were mapped onto signaling pathways for KEGG pathway analysis (Table 9 and Figure 3(d)). The differentially expressed genes were affected for small cell lung cancer, axon guidance, Fc gamma R-mediated phagocytosis, MAPK signaling pathway, focal adhesion, insulin resistance, and metabolic pathways. The results revealed that the differentially expressed gene annotation for metabolic pathway and MAPK signaling pathway are the top 2 signaling pathways, while the small lung cancer is the most significant.

### 3.5. Validation of lncRNA Expression by RT-qPCR

**Figure 4:**
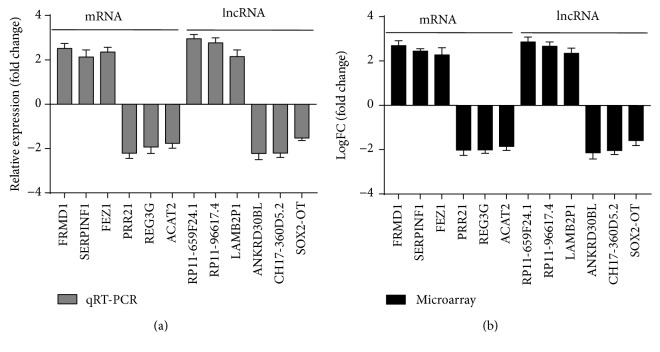
Validation of mRNA and lncRNA expression by RT-qPCR. The RT-qPCR validation of six mRNAs and lncRNAs. The results showed that the expression levels of the mRNAs FRMD1, SERPINF1, and FEZ1 and lncRNAs RP11-659F24.1, RP11-966I7.4, and LAMB2P1 were upregulated compared with the controls, and the expression levels of the mRNAs PRR21, REG3G, and ACAT2 and lncRNAs ANKRD30BL, CH17-360D5.2, and SOX2-OT were downregulated compared with the controls (a). The results of RT-qPCR were similar to those obtained from the microarray analysis (b).

To validate the up- and downregulation of Cyr61 changes of mRNA and lncRNA expression detected by microarray, six mRNAs (FRMD1, SERPINF1, FEZ1, PRR21, REG3G, and ACAT2) and six lncRNAs (RP11-659F24.1, RP11-966I7.4, LAMB2P1, ANKRD30BL, CH17-360D5.2, and SOX2-OT) were selected. Their expression was examined with RT-qPCR. We observed a good agreement across the two methods. The results of the RT-qPCR were similar to those obtained from the microarray. In both microarrays and RT-qPCR, the expression levels of the mRNAs FRMD1, SERPINF1, and FEZ1 and lncRNAs RP11-659F24.1, RP11-966I7.4, and LAMB2P1 were upregulated, and the expression levels of the mRNAs PRR21, REG3G, and ACAT2 and lncRNAs ANKRD30BL, CH17-360D5.2, and SOX2-OT were downregulated after knocking down the Cyr61 gene. This validation indicated good reproducibility and reliability of the observed changes in expression detected by mRNA and lncRNA microarrays (Figure 4).

### 3.6. Downregulation Cyr61 Promotes Proliferation of HEK293T Cells

**Figure 5:**
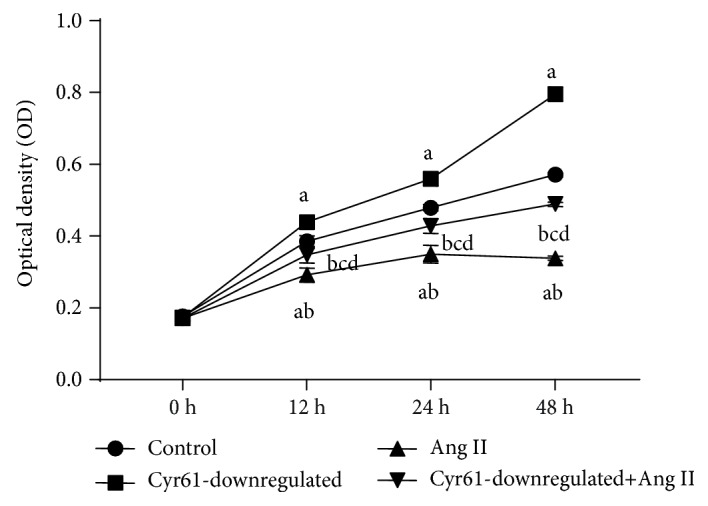
Effects of Cyr61 on Ang II-induced proliferation in HEK293T cells. To investigate the effects of Cyr61 on Ang II-induced proliferation in HEK293T cells, the HEK293T cells were treated with Ang II after knocking down the Cyr61 gene. Proliferation was estimated using Cell Counting Kit-8 assay. ^a^*P* < 0.05 compared with controls, ^b^*P* < 0.05 compared with Cyr61 knockdown, ^c^*P* < 0.05 compared with Ang II without Cyr61 knockdown, and ^d^*P* > 0.05 compared with controls. The data represent means ± SD from 3 independent experiments.

To investigate the role of Cyr61 knockdown on the growth of HEK293T cell lines, we treated the HEK293T cells with Ang II at various time points (0, 12, 24, and 48 h) after knocking down the Cyr61 gene and then performed the CCK-8 assay to examine the proliferation of HEK293T cells. Knockdown of the Cyr61 gene increased the cell proliferation of HEK293T cells significantly, and Ang II inhibited proliferation when compared with control groups. The proliferation rate of Ang II with Cyr61 knockdown groups was significantly increased compared to cells treated with Ang II without Cyr61 knockdown. There was no significant difference between controls and Ang II with Cyr61 knockdown (Figure 5). Taken together, these data suggest a pro-proliferative role of downregulation of the Cyr61 gene and an antiproliferative role of Ang II in HEK293T cells.

### 3.7. Downregulation of Cyr61 Inhibits Apoptosis of HEK293T Cells

**Figure 6:**
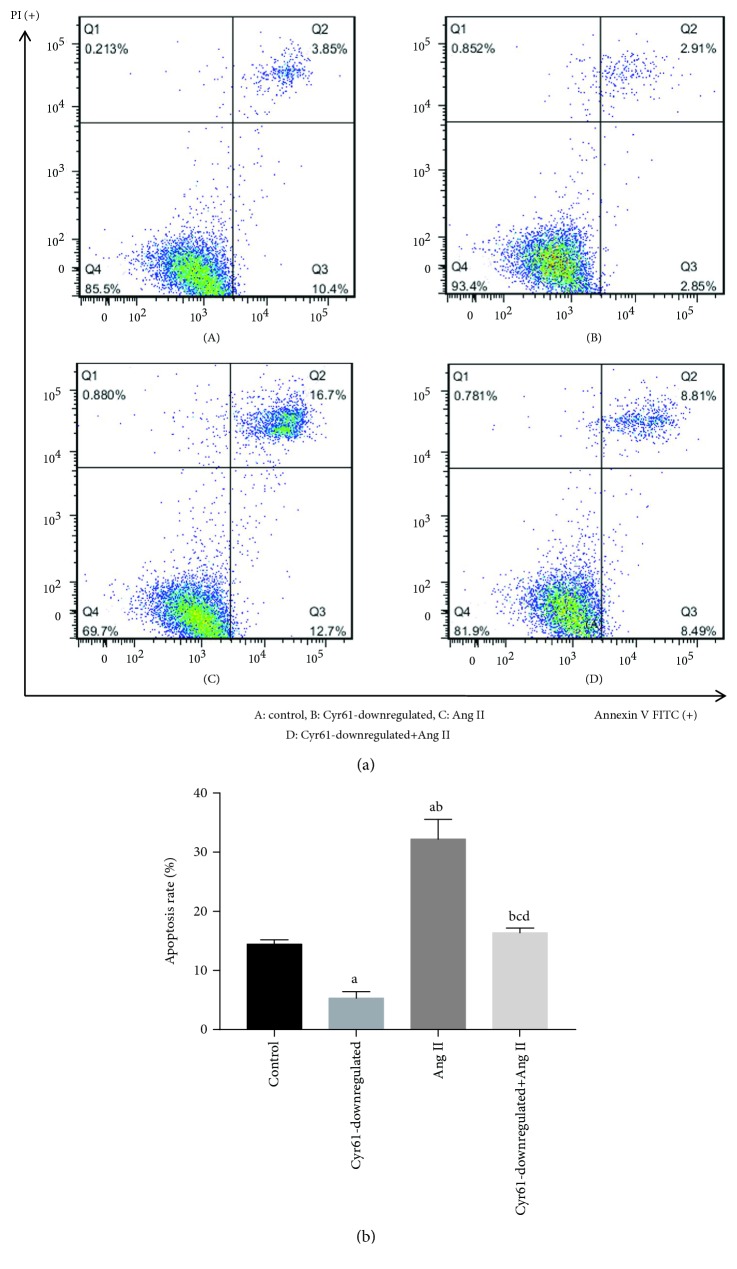
Effects of Cyr61 on Ang II-induced apoptosis in HEK293T cells. To investigate the effects of Cyr61 on Ang II-induced apoptosis on HEK293T cells (a). the HEK293T cells were treated with Ang II after knocking down the Cyr61 gene for 48 h, and apoptosis was measured by Annexin V-FITC/PI staining and flow cytometry. (b). The ratio of apoptotic cells to the total number of cells. The number of apoptotic cells equals the sum of the cells in the Q2 (early-stage cell apoptosis rate) and Q3 (late-stage cell apoptosis rate). ^a^*P* < 0.05 compared with controls, ^b^*P* < 0.05 compared with Cyr61 knockdown, ^c^*P* < 0.05 compared with Ang II without Cyr61 knockdown, and ^d^*P* > 0.05 compared with controls. The data represent means ± SD from 3 independent experiments.

In order to investigate the role of Cyr61 on the apoptosis of HEK293T cells, we treated the HEK293T cells with Ang II after knocking down the Cyr61 gene for 48 h, and then cells were stained with Annexin V-FITC and PI, followed by flow cytometry analysis. Under both conditions, the apoptosis rate was decreased significantly in Cyr61 knockdown groups (5.14 ± 1.04%) and significant cell apoptosis was observed in the Ang II groups (32.50 ± 2.95%) compared with controls (12.92 ± 2.07%). The percentage of Annexin V-FITC for Ang II with Cyr61 knockdown groups were significantly decreased (16.19 ± 1.40%) compared with Ang II without Cyr61 knockdown (32.50 ± 2.95%). However, there was no significant difference between controls (12.92 ± 2.07%) and Ang II with Cyr61 knockdown (16.19 ± 1.40%; Figure 6). Taken together, these data suggest a proliferative role after downregulation of the Cyr61 gene and an antiproliferative role of Ang II in HEK293T cells.

### 3.8. Downregulation of Cyr61 Promotes Cell Cycle Progression of HEK293T Cells

**Figure 7:**
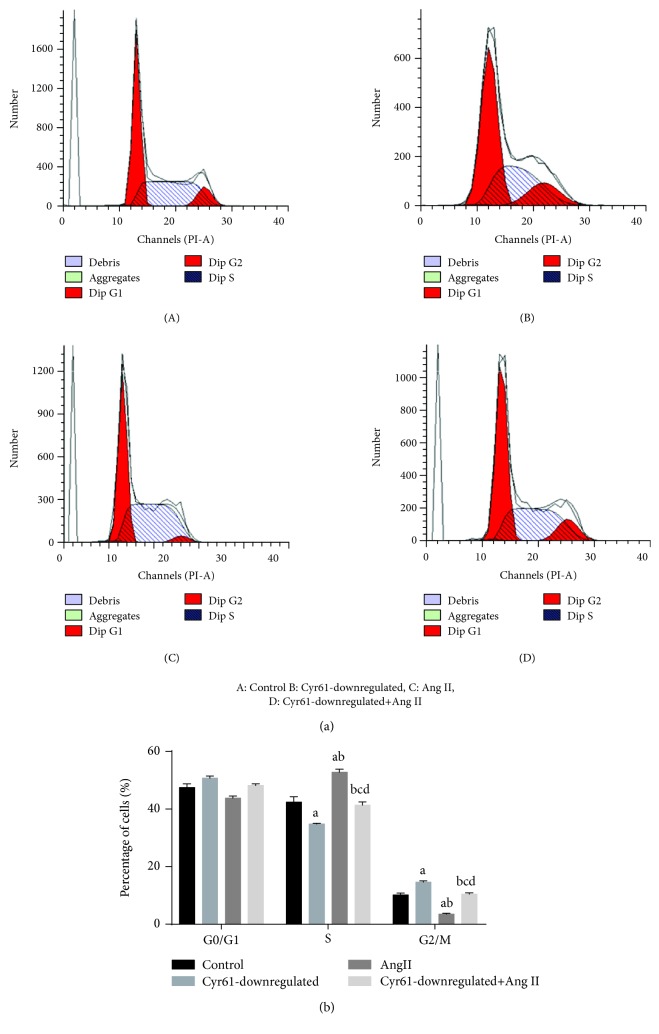
Effects of Cyr61 on Ang II-induced cell cycle progression in HEK293T cells. To investigate the effects of Cyr61 on Ang II-induced cycle progression in HEK293T cells, the HEK293T cells were treated with Ang II after knocking down the Cyr61 gene for 48 h. Cells were stained with PI and collected to be examined on a flow cytometer. ^a^*P* < 0.05 compared with controls, ^b^*P* < 0.05 compared with Cyr61 knockdown, ^c^*P* < 0.05 compared with Ang II without Cyr61 knockdown, and ^d^*P* > 0.05 compared with controls. The data represent means ± SD from 3 independent experiments.

Having found the interaction between Cyr61 and Ang II on the apoptosis and proliferation in HEK293T cells, the interaction between these on the cell cycle was investigated by treating HEK293T cells with Ang II for 48 h after knocking down the Cyr61 gene. Cells were stained with PI and collected to be examined on a flow cytometer. An increase in the percentage of cells in the G2/M phase (14.57 ± 0.51%) and a marked decrease in the percentage of cells in the S phase (34.74 ± 0.30%) in Cyr61 knockdown groups compared with the other groups were seen. In addition, a decrease in the percentage of cells in the G2/M phase (3.45 ± 0.39%) and a marked increase in the percentage of cells in the S phase (52.78 ± 1.11%) in Ang II groups were also seen. There was no significant difference between controls (G2/M phase: 10.16 ± 0.63%, S phase: 42.36 ± 1.88%) and Ang II with Cyr61 knockdown groups (G2/M phase: 10.42 ± 0.47%, S phase: 41.40 ± 1.01%; Figure 7). Downregulation of Cyr61 promoted the cell cycle progression, and treatment with Ang II inhibited the cell cycle progression.

### 3.9. Ang II Promotes Cyr61 Expression in HEK293 T Cells

**Figure 8:**
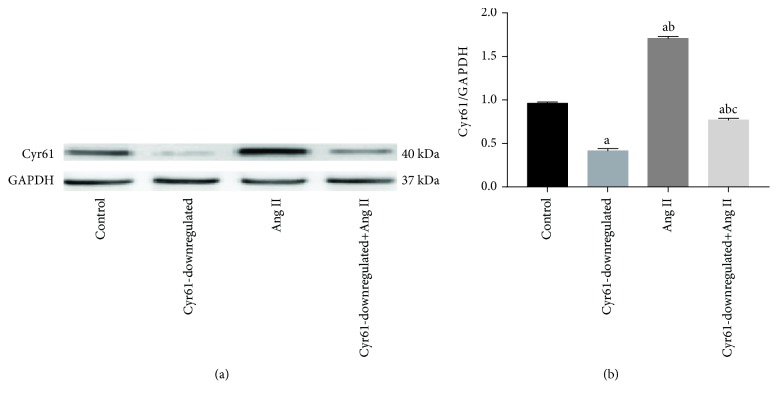
The expression of Cyr61 protein treated with Ang II. To investigate whether Ang II induces Cyr61 expression in 293 cells, we incubated HEK293T cells for 48 hours in DMEM with or without Ang II. (a). Western blots were performed to detect the expression levels of *GAPDH and* Cyr61 protein in HEK293T cells. (b) Quantitative analysis of Cyr61 protein. *GAPDH* was used as internal control. Statistical analysis was performed using one-way ANOVA. ^a^*P* < 0.05 compared with controls, ^b^*P* < 0.05 compared with Cyr61 knockdown, and ^c^*P* < 0.05 compared with Ang II without Cyr61 knockdown. The data represent means ± SD from 3 independent experiments.

To investigate whether Ang II induces Cyr61 expression in 293 cells, we incubated HEK293T cells for 48 hours in DMEM with or without Ang II. Cyr61 transcript levels as determined by western blots were elevated (1.56 ± 0.031, Cyr61/GAPDH) in HEK293T cells exposed to Ang II compared with controls (0.98 ± 0.026, Cyr61/GAPDH). Furthermore, the Cyr61 protein transcript levels of HEK293T cells which had the Cyr61 gene knocked down was elevated significantly (0.72 ± 0.027, Cyr61/GAPDH) after treating with Ang II compared to cells not treated with Ang II (0.48 ± 0.032, Cyr61/GAPDH; Figure 8).

## 4. Discussion

To date, the CRISPR/Cas9 system is known as a molecular scissor and is widely used in various studies, including cancer research, drug discovery, treatment of mental disease, and applications in plant husbandry [35]. A limitation of this method is that the Cas9 construct is either permanently integrated or must be later removed with a subsequent reagent delivery and/or clonal selection step to achieve editing without scarring [36, 37]. In this study, we knocked down Cyr61 via CRISPR/Cas9 plasmid cotransfection with the Cyr61 HDR plasmid. Cyr61 HDR utilizes template DNA (usually the homologous chromosome) to repair DNA in a precise or “error-free” manner [38]. Cyr61 HDR plasmid cotransfected with Cyr61 CRISPR/Cas9 KO plasmid and was designed for repair of the site-specific Cas9-induced DNA cleavage within the Cyr61 gene. During repair, the Cyr61 HDR plasmid incorporates a puromycin resistance gene to enable selection of stable knockout cells and an RFP (red fluorescent protein) gene to visually confirm that the transfection has occurred. Using nonintegrated plasmid vectors that express a puromycin N-acetyltransferase (PAC) gene, whose expression and translation is linked to that of Cas9, we selected for cells based on their early expression levels of Cas9 protein. Edited cells isolated using this method did not contain any detectable off-target mutations and displayed expected functional phenotypes after directed differentiation [38].

Cyr61 is a secreted, extracellular matrix- (ECM-) associated signaling protein of the CCN family [11]. Cyr61 is capable of regulating a broad range of cellular activities, including cell adhesion, migration, proliferation, differentiation, apoptosis, and senescence through interaction with cell surface integrin receptors and heparan sulfate proteoglycans. During embryonic development, Cyr61 is critical for cardiac septal morphogenesis, blood vessel formation in placenta, and vascular integrity. In adulthood, Cyr61 plays important roles in inflammation and tissue repair and is associated with diseases related to chronic inflammation, including rheumatoid arthritis, atherosclerosis, diabetes-related nephropathy and retinopathy, and many different forms of cancers.

lncRNAs are defined as transcripts longer than 200 nucleotides [39]. It is estimated that at least 90% of RNAs transcribed by the human genome are lncRNAs [40] and was previously considered as “junk gene” or transcriptional “noise” [41]. Although the function of lncRNA remains unclear, in recent years, numerous studies have found that lncRNAs have important biological functions; regulate gene expression via epigenetic regulation, transcriptional regulation, and transcriptional regulation [42]; and participate in embryonic development, cell proliferation, differentiation, apoptosis, and several other biological processes. lncRNAs also play important roles in modulating the innate and adaptive immune responses and immune cell development [43, 44]. Moreover, emerging evidence suggests that lncRNAs play a decisive role in such autoimmune diseases as systemic lupus erythematosus (SLE), rheumatoid arthritis (RA), type 1 diabetes mellitus (T1DM), multiple sclerosis (MS) [45], autism spectrum disorders [46], tumor biology [47, 48], and thoracic and abdominal aortic aneurysms [49]. Future research that deepens our understanding of the biological roles and properties of lncRNAs will enable researchers to harness their potential as valuable diagnostic and therapeutic targets for human diseases.

Unfortunately, until now, Cyr61 functional recovery highlighting its transcriptional networks has not been reported and lncRNAs may play an important regulatory role and possibly mediate Cyr61-involved diseases. To explore the molecular mechanisms involved in Cyr61-induced changes of cell functions, in this study, high-throughput microarray technology designed for genome-wide identification was performed to detect the profiles of aberrantly expressed lncRNAs and mRNAs in downregulated Cyr61 HEK293 cells. A total of 23184 lncRNAs and 28264 mRNAs were normalized. 26 lncRNAs and 212 mRNAs were upregulated, and 74 lncRNAs and 233 mRNAs were downregulated after Cyr61 knockdown. Analysis of the cellular components, molecular functions, biological processes, and regulatory pathways associated with the differentially expressed mRNAs revealed the *down*stream mechanisms of the Cyr61 gene. The differentially expressed genes were affected for small cell lung cancer, axon guidance, Fc gamma R-mediated phagocytosis, MAPK signaling pathway, focal adhesion, insulin resistance, and metabolic pathways.

Based on pathway analysis, thirteen major mRNAs were found to be involved in the MAPK signaling pathway. The MAPK signaling pathway is essential in regulating numerous cellular processes including inflammation, cell stress response, cell differentiation, cell division, cell proliferation, metabolism, motility, and apoptosis. The role of the MAPK pathway in cancer, immune disorders, and neurodegenerative diseases has been well recognized. The results of gene microarray analysis suggest that downregulation of Cyr61 regulates the cellular function either by regulating the signaling pathway or by regulating the expression of some others genes. This warrants further study.

The mechanisms by which overexpression of Cyr61 increases cancer risk are likely to be multifactorial. Part of the effects seen appears to be due to the direct interaction between Cyr61 protein with the MAPK signaling pathway and p53 as well as an effect on cell apoptosis [7, 50, 51]. An effect mediated by the MAPK signaling pathway is given additional support by our transcriptome analysis. In addition, based on our findings, Cyr61 may also contribute to malignant transformation indirectly through its effects on other genes, which were found to be differentially expressed in this study and have been implicated in malignant transformation. These genes including SERPINF1, CRABP2, and TXNIP [52–54]. REG3G, ACAT2, and ATF5 have also been found in various cancers [55–57]. Several of the differentially expressed lncRNAs we show in Table 4 have also been found to be associated with cancer development, including CH17-360D5.2, SOX2-OT, and HOXA11 [58–60]. These findings suggest that the effect of Cyr61 on malignant transformation is multifactorial and not simply due to a direct interaction with the MAPK signaling pathway.

Additionally, we also found that the differently expressed genes are involved in other pathophysiological processes unrelated to cancer. For mRNAs, for example, PRR21 may be involved in stress responses that are related to phosphorylation of mitochondrial proteins [61]. Mutation of PIGW is associated with West syndrome and hyperphosphatasia with mental retardation syndrome [62]. Rbm19 plays an important role in the development of intestinal epithelium cells [63]. Thus, the genetic changes associated with Cyr61 may be involved in the occurrence and development of many diseases.

GO analysis predicted that up- and downregulated mRNAs were associated with several biological processes, cellular components, and molecular functions in HEK 293 cells with downregulated Cyr61. The GO annotation indicated that these gene products were attributed to the progress of a variety of biochemical reactions, such as cell-cell adhesion, negative regulation of cell migration, protein binding, mRNA processing, cell-cell adherens junction and poly(A) RNA binding. Cell adhesion and migration are the central processes in the development and maintenance of multicellular organisms. Tissue formation during embryonic development, wound healing, and immune responses all require the orchestrated movement of cells in particular directions to specific locations. Errors during these processes may have serious consequences, including intellectual disability, vascular disease, tumor formation, and metastasis [64].

It has been reported that Cyr61 mediates numerous cellular activities, including cell migration, adhesion, apoptosis, and proliferation [65]. As a cell adhesion protein, through binding to particular integrins, Cyr61 associates with the adhesion activities of endothelial cells, fibroblast cells, smooth muscle cells, and monocytes [66–69]. Numerous studies have shown that Cyr61 is involved in cell migration, including smooth muscle cell migration, fibroblast-like synoviocyte invasion, and cancerous adenocarcinoma [70–72], and is seen as a promising therapeutic target. Nevertheless, the mechanism by which Cyr61 participates in cell adhesion and migration is still not completely clear because both cell adhesion and migration are complex biological processes. Our research shows that a large number of abnormal genes are involved in cell adhesion and migration. It is anticipated that this can provide additional research directions for Cyr61-involved diseases. Subsequently, exploration of the correlations between abnormal genes and signal pathways is critical for developing new biomarkers for the early diagnosis and therapeutic surveillance of Cyr61-involved diseases.

We also studied the correlation between Ang II and Cyr61 in HEK293 cells. It is well recognized that the RAS is an important hormonal system in humans and exists not only as a circulating and paracrine but also as an intracrine system [20, 73]. There have been many well-documented pathophysiologic functions of the intrarenal RAS. Increasing lines of evidence have indicated the RAS was involved in a variety of diseases including tumors, depression, glaucomatous, cardiovascular diseases, kidney diseases, and Parkinson's disease [74–78]. The pathological processes include fibrosis, inflammation, and immune responses, and it also induces inflammation, renal cell growth, mitogenesis, apoptosis, migration, and differentiation [79], and the adverse effects of the RAS on these diseases are due mainly to increased levels of Ang II, which exerts its effects through the classical ACE-AngII-AT1R axis [80].

In this study, we knocked down the Cyr61 gene of HEK293T cells by using the Cyr61 CRISPR/Cas9 KO plasmid and Cyr61 HDR transfection plasmid and then treated the HEK293T cells with Ang II. The cell functions of these HEK293T cells were then observed including the cell cycle, cell proliferation, and cell apoptosis. Our results showed that the expression of Cyr61 protein was significantly decreased after knocking down the Cyr61 gene by Cyr61 CRISPR/Cas9 KO plasmid and HDR transfection plasmid in HEK293T cells compared with the controls, and knockdown of the Cry61 gene can promote cell proliferation and inhibit apoptosis significantly. These results may be related to promoting the cell cycle. In this study, cell-cycle distribution analysis showed significantly decreased cell populations in the S phase and increased cell populations in the G2/M phase when the Cyr61 gene was knocked down. Our results are similar to those of some previous studies. For example, Xiong et al. reported that Ang II significantly inhibited cell survival, induced cell cycle arrest, and enhanced cell apoptosis in HK-2 cells [81].

Interestingly, the HEK293T cells with knockdown of the Cyr61 gene treated with Ang II have a higher cell proliferation rate and G2/M phase and lower apoptosis rate than the Ang II-added group of cells. We further examined the expression of Cyr61 after treating with Ang II. Consistent with the findings of Hilfiker et al. [82], we found that Cyr61 transcript levels were elevated in HEK293T cells exposed to Ang II compared with controls. Furthermore, the Cyr61 protein transcript levels of HEK293T cells with the Cyr61 gene knocked down were elevated significantly after treating with Ang II compared to cells without Ang II treatment. In contrast, the proliferation and apoptosis of Cyr61-downregulated + Ang II cells were not different to control cells, although the Cyr61 level of downregulated + Ang II cells was lower than that of the controls was. This probably means that Cyr61 is not the only factor which is involved in Ang II-induced apoptosis and antiproliferation of these cells and suggests that some others factors and signaling pathways, such as AT2 receptor (angiotensin type 2 receptor), p38 signaling pathway, and IGF-IR-PI3K-Akt signaling are also involved in these processes [83–85]. Accordingly, further research into the mechanisms of Ang II-induced apoptosis and antiproliferation is needed. Taken together, downregulation of Cyr61 is of benefit to the cell cycle and is antiapoptotic, and Ang II induces HEK293T cell injury, at least in part, by upregulating the expression of Cyr61 protein.

There are some limitations in our study. The cells transfected with the vector that expresses puromycin N-transferase (PAC) were not involved, and the unspecific effects of PAC on HEK293T cells were not observed in the present study. The puromycin selection may enhance Cas-9 expression in the transfected cells so unspecific effects by Cas-9 cannot be ruled out. lncRNAs can play their biological functions by interacting with mRNAs. The biological meanings of differentially expressed lncRNAs will be more convinced if the lncRNAs and mRNA coexpression network can be performed between the validated lncRNAs and their related mRNA based on the correlation analysis.

## 5. Conclusions

*Cyr61* is mutated in a variety of diseases. We report for the first time that many lncRNAs and mRNAs are significantly upregulated or downregulated and that specific metabolic pathways may play important roles in the *down*stream events of the Cyr61 gene. The significantly altered lncRNAs and mRNAs levels may be related to many pathological processes. It appears that Cyr61 is involved in Ang II-induced injury in HEK293T cells. It is envisaged that studies of the functional mechanisms of these differentially expressed lncRNAs and mRNAs as well as exploration of the pathways involved will provide novel targets for Cyr61-involved diseases.

## Figures and Tables

**Table 1:** The guide RNAs sequences.

gRNA	Name	Sequence
Cyr61-gRNA1	Cyr61-A	GTTGTCATTGGTAACTCGTG
Cyr61-gRNA2	Cyr61-B	ATGCGGTTCCGCTGCGAAGA
Cyr61-gRNA3	Cyr61-C	CAAGTACTGCGGTTCCTGCG

**Table 2:** The RNA quality test results.

Sample name	Sample code	OD_260_/OD_280_ ≥ 1.8	OD_260_/OD_230_ ≥ 1.5	Total of RNA	RIN>6	Result
2402-1	1H617022202-01A	2.02	2.12	53.6	10.0	Pass
2402-2	1H617022202-02A	2.02	2.02	55.8	10.0	Pass
2402-3	1H617022202-03A	2.01	2.15	57.0	10.0	Pass
2403-1	1H617022202-04A	2.01	2.12	66.5	9.9	Pass
2403-2	1H617022202-05A	2.01	2.07	71.4	10.0	Pass
2403-3	1H617022202-06A	2.02	2.13	64.6	9.9	Pass

RIN values were detected using the Agilent RNA 6000 Nano Assay, and the integrity of 18s and 28s (for eukaryotes) in the RNA samples was detected to determine the integrity of RNA. RIN is between 0 and 10, with a full score of 10. RIN values of more than 6 indicate good sample integrity and can be used in ChIP experiments.

**Table 3:** Primers for qRT-PCR.

Name	Forward primer (5′-3′)	Reverse primer (5′-3′)
GAPDH	AACTTTGGCATTGTGGAAGG	GGATGCAGGGATGATGTTCT
RP11-659F24.1	CTCGGCTCACTGAAAACTCC	TTCAACACCGTGCCTTCATA
RP11-966I7.4	TCCTGGGGCACTAATAGCAG	TCTTACGAGCGTCTCCACCT
LAMB2P1	CCTTTCTGGTTCGGCCTCAG	GGATCCTCTCAGGGATGACA
ANKRD30BL	CCAGAAGGAACATCTACAGGAACACC	CAAGCGTGCAGCCTCGTCAG
CH17-360D5.2	CCGCCATACAGATACGAAGCCAAG	ATCCTCTCCAGGTAGCCACTTGTG
SOX2-OT	AGGTCTTGGAGGCTGGTGTAAGG	ACCATGTGAAGGAGCTTGCAGTTC
FRMD1	GCACTACGTGGAAAACGGAAG	GAGTGTGGCTCGAAGTACCTC
SERPINF1	TTCAAAGTCCCCGTGAACAAG	GAGAGCCCGGTGAATGATGG
FEZ1	CCACTGGTGAGTCTGGATGAA	CGGAAGAAAAATTCTCAAGCTCG
PRR21	CTCCGCGTTCTTATCTGTG	GGACAGGAGGCTGAGAAGTT
REG3G	GGTGAGGAGCATTAGTAACAGC	CCAGGGTTTAAGATGGTGGAGG
ACAT2	GCGGACCATCATAGGTTCCTT	ACTGGCTTGTCTAACAGGATTCT

**Table 4:** The top 10 most significant up- and downregulated lncRNAs.

*P* value	Gene symbol	Fold change (log FC)		Gene symbol	*P* value
		Upregulation	Downregulation		
0.0062	RP11-659F24.1	2.8653	-2.1347	ANKRD30BL	0.0026
0.0496	RP11-966I7.4	2.6712	-2.0459	CH17-360D5.2	0.0015
2.44*E* − 06	LAMB2P1	2.3529	-1.5879	SOX2-OT	0.0010
1.14*E* − 05	CECR5-AS1	1.8255	-1.5293	CTD-3099C6.11	0.0125
5.98*E* − 06	RP11-617F23.1	1.7205	-1.5262	RP11-227G15.3	0.0324
7.29*E* − 06	RP11-49I11.1	1.5183	-1.5095	RP1-290I10.3	0.0011
3.75*E* − 05	LINC00505	1.4474	-1.5090	HOXA11	1.25*E* − 05
0.0005	LINC01021	1.4186	-1.5085	FLJ33534	0.0024
2.64*E* − 05	RP11-395B7.7	1.3960	-1.4651	LINC00948	0.0190
0.0003	LINC01021	1.3026	-1.4335	DLEU2	2.26*E* − 05

**Table 5:** The top 10 most significant up- and downregulated mRNAs.

*P* value	Gene symbol	Fold change (log FC)		Gene symbol	*P* value
		Upregulation	Downregulation		
5.10*E* − 07	FRMD1	2.6979	-2.020	PRR21	0.0039
4.62*E* − 07	SERPINF1	2.4507	-2.008	REG3G	0.0022
4.47*E* − 06	FEZ1	2.2722	-1.851	ACAT2	0.0006
1.50*E* − 06	CRABP2	2.2282	-1.701	PIGW	3.57*E* − 05
6.45*E* − 07	G0S2	2.2042	-1.698	RBM19	0.0019
4.76*E* − 06	CDA	2.1142	-1.674	RPL27A	6.55*E* − 06
1.29*E* − 06	TXNIP	2.0500	-1.623	ATF5	7.15*E* − 06
9.71*E* − 06	PADI2	2.0345	-1.610	ADI1	2.93*E* − 05
0.0001	SERPINF1	2.0129	-1.581	ADI1	7.22*E* − 06
1.27*E* − 05	CRABP2	1.9778	-1.565	UBAP2L	0.0138

**Table 6:** The top 10 biological process terms with the highest *P* values.

GO ID	Gene set name	Count	*P* value
GO:0030336	Negative regulation of cell migration	9	0.0009
GO:0045892	Negative regulation of transcription, DNA-templated	22	0.0023
GO:0006488	Dolichol-linked oligosaccharide biosynthetic process	4	0.0036
GO:0071333	Cellular response to glucose stimulus	6	0.0048
GO:0006397	mRNA processing	11	0.0049
GO:0098609	Cell-cell adhesion	14	0.0053
GO:0000381	Regulation of alternative mRNA splicing, via spliceosome	5	0.0083
GO:0048843	Negative regulation of axon extension involved in axon guidance	4	0.0171
GO:0045010	Actin nucleation	3	0.0180
GO:0006405	RNA export from nucleus	5	0.0290

**Table 7:** The top 10 cellular components with the highest *P* values.

Go ID	Geneset name	Count	*P* value
GO:0005829	Cytosol	102	1.09*E* − 05
GO:0016020	Membrane	73	3.07*E* − 05
GO:0005730	Nucleolus	34	0.0004
GO:0005634	Nucleus	139	0.0012
GO:0005654	Nucleoplasm	78	0.0029
GO:0005913	Cell-cell adherens junction	14	0.0158
GO:0005737	Cytoplasm	126	0.0201
GO:0005604	Basement membrane	6	0.0227
GO:0005783	Endoplasmic reticulum	26	0.0330
GO:0032040	Small-subunit processome	4	0.0341

**Table 8:** The top 10 molecular functions terms with the highest *P* values.

Go ID	Geneset name	Count	*P* value
GO:0044822	Poly(A) RNA binding	51	7.30*E* − 07
GO:0005515	Protein binding	224	0.0001
GO:0003714	Transcription corepressor activity	14	0.0004
GO:0008134	Transcription factor binding	15	0.0033
GO:0019899	Enzyme binding	16	0.0055
GO:0003723	RNA binding	22	0.0074
GO:0098641	Cadherin binding involved in cell-cell adhesion	14	0.0098
GO:0001228	Transcriptional activator activity, RNA polymerase II transcription regulatory region sequence-specific binding	7	0.0162
GO:0044212	Transcription regulatory region DNA binding	11	0.0165
GO:0016887	ATPase activity	10	0.0170

**Table 9:** The top 7 molecular signaling pathways for KEGG pathway analysis with the highest *P* values.

Pathway ID	Definition	Count	*P* value
Hsa05222	Small cell lung cancer	8	0.0032
Hsa04360	Axon guidance	9	0.0084
Hsa04666	Fc gamma R-mediated phagocytosis	7	0.0122
Hsa04010	MAPK signaling pathway	13	0.0136
Hsa04510	Focal adhesion	11	0.0194
Hsa04931	Insulin resistance	7	0.0369
Hsa01100	Metabolic pathways	38	0.0415

## Data Availability

The data used to support the findings of this study are available from the corresponding author upon request.
